# Supplementary material for: Neural substrates, dynamics and thresholds of galvanic vestibular stimulation in the behaving primate
Source: Nat Commun. 2019 Apr 23;10:1904. doi: 10.1038/s41467-019-09738-1 (PMC6478681; doi:10.1038/s41467-019-09738-1)
Supplement: Supplementary file 1 — Supplementary information [file 41467_2019_9738_MOESM1_ESM.pdf]

Supplementary Information

Neural Substrates, Dynamics and Thresholds of Galvanic Vestibular Stimulation in the Behaving Primate

Annie Kwan[1], Patrick A. Forbes[2,3,4], Diana E. Mitchell[5], Jean-Sébastien Blouin[4], and Kathleen E. Cullen[*5,6]

[1]Department of Biomedical Engineering, McGill University, Montreal, QC, H3G 1Y6, Canada
[2]Department of Neuroscience, Erasmus MC, University Medical Center Rotterdam, Rotterdam, 3000 CA, The Netherlands
[3]Department of BioMechanical Engineering, Delft University of Technology, Delft, 2628 CD, The Netherlands
[4]School of Kinesiology, University of British Columbia, Vancouver, BC, V6T 1Z1, Canada
[5]Department of Physiology, McGill University, Montreal, QC, H3G 1Y6, Canada
[6]Department of Biomedical Engineering, Johns Hopkins University, Baltimore, Maryland, USA

Correspondence: kathleen.cullen@jhu.edu

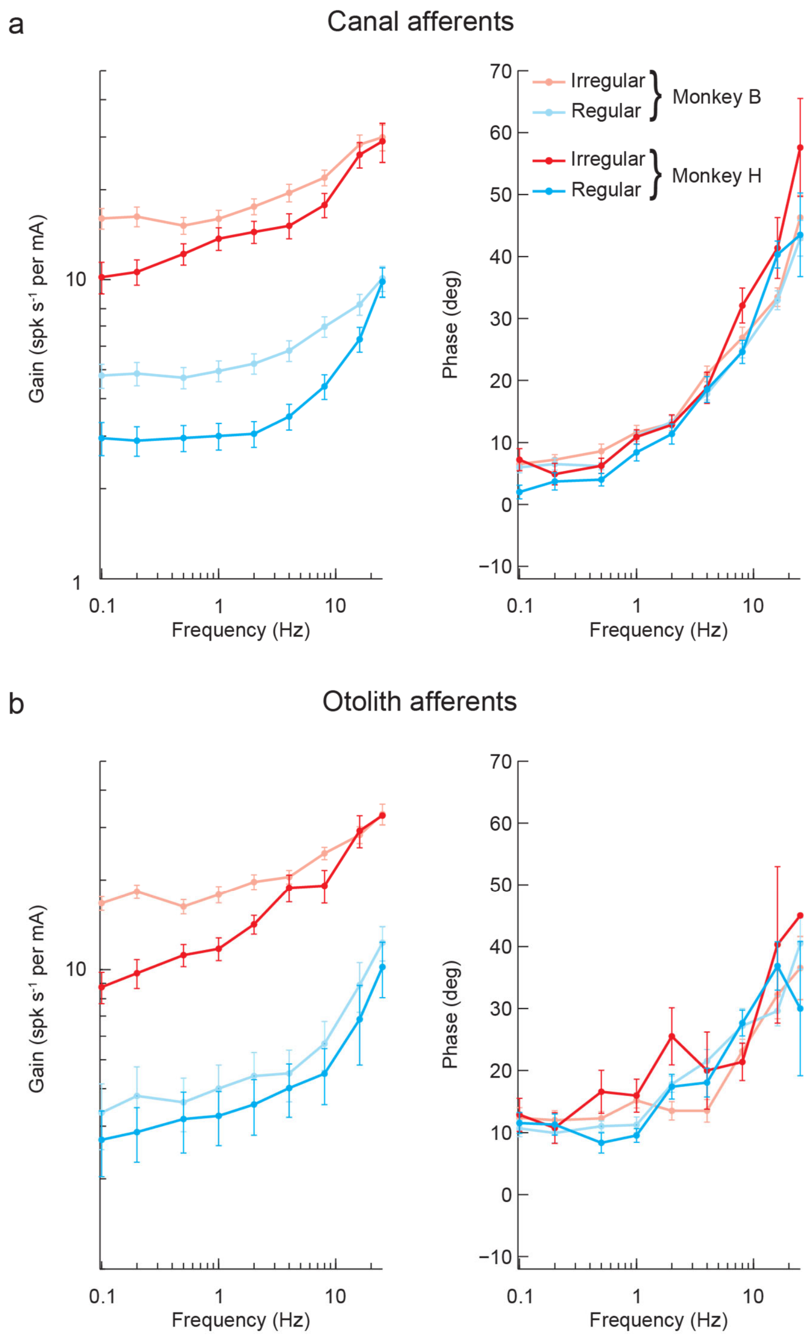

Supplementary Figure 1. Comparison of vestibular afferents recorded from two animals. (a) Population averaged gain and phase of regular (Monkey B: light blue; Monkey H: dark blue) and irregular (Monkey B: light red; Monkey H: dark red) canal afferents to sinusoidal GVS. (b) Population averaged gain and phase of regular (Monkey B: light blue; Monkey H: dark blue) and irregular (Monkey B: light red; Monkey H: dark red) otolith afferents to sinusoidal GVS.

Supplementary Table 1. Statistics summary table. Grey shaded p-values represent effects that were not significant

Figure	Panel	Test used	n		Descriptive statistics shown	P value	Degree of freedom & F/t values
			Exact value	Definition			
1	**C** left	Repeated measures ANOVA	15	Gain values from 3 monkeys across 5 different frequencies	Error bars are mean ± s.e.m.	p = 0.604 for frequency factor	$F_{4,8}$ = 0.72 for frequency factor;
	**C** right	Repeated measures ANOVA	15	Phase values from 3 monkeys across 5 different frequencies	Error bars are mean ± s.e.m.	*p < 0.001 for frequency factor*	$F_{4,8}$ = 59.25 for frequency factor;
	**D** left	Repeated measures ANOVA	15	Amplitude values from 3 monkeys across 5 different current amplitudes	Error bars are mean ± s.e.m.	*p = 0.002 for amplitude factor*	$F_{4,8}$ = 12.17 for amplitude factor;
	**D** right	Repeated measures ANOVA	15	Phase values from 3 monkeys across 5 different current amplitudes	Error bars are mean ± s.e.m.	p = 0.714 for amplitude factor	$F_{4,8}$ = 0.53 for amplitude factor;
2	**D** left	Two-tailed Student's t-tests with Bonferroni correction for multiple comparison at each frequency (threshold p = 0.0055)	881	Gain values from 119 canal afferents (56 irregular and 63 regular) across 9 frequencies	Error bars are mean ± s.e.m.	*0.1Hz: p < 0.001*<br>*0.2Hz: p < 0.001*<br>*0.5Hz: p < 0.001*<br>*1Hz: p < 0.001*<br>*2Hz: p < 0.001*<br>*4Hz: p < 0.001*<br>*8Hz: p < 0.001*<br>*16Hz: p < 0.001*<br>*25Hz: p < 0.001*	0.1Hz: t(75) = 9.3<br>0.2Hz: t(78) = 10.4<br>0.5Hz: t(114)=13.0<br>1Hz: t(116) = 13.1<br>2Hz: t(116) = 14.3<br>4Hz: t(116) = 12.8<br>8Hz: t(108) = 13.3<br>16Hz: t(87) = 11.9<br>25Hz: t(53) = 7.6
		Two-tailed Student's t-test (0.1 vs. 25 Hz)	132	Gain values from 132 canal afferent recordings (66 irregular and 66 regular)	Error bars are mean ± s.e.m.	*Irregular: p < 0.001*<br>*Regular: p < 0.001*	Irregular: t(64) = 6.5<br>Regular: t(64) = 7.9
	**D** right	Two-tailed Student's t-tests with Bonferroni correction for multiple comparison at each frequency (threshold p = 0.0055)	881	Phase values from 119 canal afferents (56 irregular and 63 regular) across 9 frequencies	Error bars are mean ± s.e.m.	0.1Hz: p = 0.010<br>0.2Hz: p = 0.138<br>0.5Hz: p = 0.006<br>1Hz: p = 0.094<br>2Hz: p = 0.335<br>4Hz: p = 0.071<br>8Hz: p =.022<br>16Hz: p = 0.459<br>25Hz: p = 0.091	0.1Hz: t(75) = 2.4<br>0.2Hz: t(78) = 1.10<br>0.5Hz: t(114)=2.6<br>1Hz: t(116) = 1.3<br>2Hz: t(116) = 0.43<br>4Hz: t(116) = 1.5<br>8Hz: t(108) = 2.0<br>16Hz: t(87) = 0.1<br>25Hz: t(53) = 1.4
		Two-tailed Student's t-test (0.1 vs. 25 Hz)	132	Phase values from 132 canal afferent recordings (66 irregular and 66 regular)	Error bars are mean ± s.e.m.	*Irregular: p < 0.001*<br>*Regular: p < 0.001*	Irregular: t(64) = 14.3<br>Regular: t(64) = 16.0

	**E** left	Two-tailed Student's t-tests with Bonferroni correction for multiple comparison at each frequency<br>(threshold p = 0.0055)	588	Gain values from 84 otolith afferents (54 irregular and 30 regular) across 9 frequencies	Error bars are mean ± s.e.m.	*0.1Hz: p < 0.001*<br>*0.2Hz: p < 0.001*<br>*0.5Hz: p < 0.001*<br>*1Hz: p < 0.001*<br>*2Hz: p < 0.001*<br>*4Hz: p < 0.001*<br>*8Hz: p < 0.001*<br>*16Hz: p < 0.001*<br>*25Hz: p < 0.001*	0.1Hz: t(49) = 8.7<br>0.2Hz: t(53) = 9.1<br>0.5Hz: t(80)=10.6<br>1Hz: t(82) = 9.4<br>2Hz: t(79) = 10.8<br>4Hz: t(77) = 12.0<br>8Hz: t(70) = 11.9<br>16Hz: t(53) = 8.1<br>25Hz: t(27) = 6.4
		Two-tailed Student's t-test (0.1 vs. 25 Hz)	80	Gain values from 80 otolith afferent recordings (47 irregular and 33 regular)	Error bars are mean ± s.e.m.	*Irregular: p < 0.001*<br>*Regular: p < 0.001*	Irregular: t(45) = 8.4<br>Regular: t(31) = 7.4
	**E** right	Two-tailed Student's t-tests with Bonferroni correction for multiple comparison at each frequency<br>(threshold p = 0.0055)	588	Phase values from 84 otolith afferents (54 irregular and 30 regular) across 9 frequencies	Error bars are mean ± s.e.m.	0.1Hz: p = 0.214<br>0.2Hz: p = 0.311<br>0.5Hz: p = 0.026<br>*1Hz: p = 0.002*<br>2Hz: p = 0.254<br>4Hz: p = 0.022<br>8Hz: p = 0.048<br>16Hz: p = 0.383<br>25Hz: p = 0.464	0.1Hz: t(49) = 0.8<br>0.2Hz: t(53) = 0.5<br>0.5Hz: t(80)=2.0<br>1Hz: t(82) = 3.0<br>2Hz: t(79) = 0.7<br>4Hz: t(77) = 2.0<br>8Hz: t(70) = 1.7<br>16Hz: t(53) = 0.3<br>25Hz: t(27) = 0.1
		Two-tailed Student's t-test (0.1 vs. 25 Hz)	80	Phase values from 80 otolith afferent recordings (47 irregular and 33 regular)	Error bars are mean ± s.e.m.	*Irregular: p < 0.001*<br>*Regular: p < 0.001*	Irregular: t(45) = 5.9<br>Regular: t(31) = 8.2
**4**	**B** left (red)	Two-tailed Student's t-tests with Bonferroni correction for multiple comparison at each frequency<br>(threshold p = 0.01)	357	Normalized gain values from irregular canal afferents in response to GVS or motion across different frequencies	Error bars are mean ± s.e.m.	*1Hz: p = 0.004*<br>*2Hz: p < 0.001*<br>*4Hz: p < 0.001*<br>*8Hz: p < 0.001*<br>*16Hz: p < 0.001*	1Hz: t(74) = 3.0<br>2Hz: t(73) = 7.4<br>4Hz: t(73) = 10.8<br>8Hz: t(70) = 7.1<br>16Hz: t(57) = 5.7
	**B** left (blue)	Two-tailed Student's t-tests with Bonferroni correction for multiple comparison at each frequency<br>(threshold p = 0.01)	411	Normalized gain values from regular canal afferents in response to GVS or motion across different frequencies	Error bars are mean ± s.e.m.	1Hz: p = 0.926<br>2Hz: p = 0.011<br>*4Hz: p < 0.001*<br>*8Hz: p < 0.001*<br>*16Hz: p < 0.001*	1Hz: t(84) = 0.1<br>2Hz: t(85) = 2.6<br>4Hz: t(84) = 5.8<br>8Hz: t(80) = 5.7<br>16Hz: t(68) = 5.2

	**B** right (red)	Two-tailed Student's t-tests with Bonferroni correction for multiple comparison at each frequency (threshold p = 0.0083)	433	Phase values from irregular canal afferents in response to GVS or motion across different frequencies	Error bars are mean ± s.e.m.	*0.5Hz: p < 0.001*<br>*1Hz: p < 0.001*<br>*2Hz: p < 0.001*<br>*4Hz: p < 0.001*<br>*8Hz: p < 0.001*<br>*16Hz: p < 0.001*	0.5Hz: t(74) = 7.9<br>1Hz: t(74) = 9.9<br>2Hz: t(73) = 9.3<br>4Hz: t(73) = 8.5<br>8Hz: t(70) = 8.5<br>16Hz: t(57) = 7.0
	**B** right (blue)	Two-tailed Student's t-tests with Bonferroni correction for multiple comparison at each frequency (threshold p = 0.0083)	498	Phase values from regular canal afferents in response to GVS or motion across different frequencies	Error bars are mean ± s.e.m.	*0.5Hz: p < 0.001*<br>*1Hz: p < 0.001*<br>*2Hz: p < 0.001*<br>*4Hz: p < 0.001*<br>*8Hz: p < 0.001*<br>*16Hz: p < 0.001*	0.5Hz: t(85) = 4.5<br>1Hz: t(84) = 2.9<br>2Hz: t(85) = 3.8<br>4Hz: t(84) = 5.8<br>8Hz: t(80) = 9.7<br>16Hz: t(68) = 9.6
**5**	**B** left (red)	Two-tailed Student's t-tests with Bonferroni correction for multiple comparison at each frequency (threshold p = 0.01)	320	Normalized gain values from irregular otolith afferents in response to GVS or motion across different frequencies	Error bars are mean ± s.e.m.	1Hz: p = 0.76<br>2Hz: p = 0.96<br>4Hz: p = 0.11<br>*8Hz: p < 0.001*<br>*16Hz: p < 0.001*	1Hz: t(68) = 0.3<br>2Hz: t(66) = 0.1<br>4Hz: t(64) = 1.6<br>8Hz: t(61) = 3.6<br>16Hz: t(51) = 7.8
	**B** left (blue)	Two-tailed Student's t-tests with Bonferroni correction for multiple comparison at each frequency (threshold p = 0.01)	224	Normalized gain values from regular otolith afferents in response to GVS or motion across different frequencies	Error bars are mean ± s.e.m.	1Hz: p = 0.53<br>2Hz: p = 0.21<br>4Hz: p = 0.22<br>8Hz: p = 0.16<br>16Hz: p = 0.03	1Hz: t(46) = 0.6<br>2Hz: t(46) = 1.3<br>4Hz: t(45) = 1.2<br>8Hz: t(42) = 1.5<br>16Hz: t(35) = 2.2
	**B** right (red)	Two-tailed Student's t-tests with Bonferroni correction for multiple comparison at each frequency (threshold p = 0.0083)	389	Phase values from irregular otolith afferents in response to GVS or motion across different frequencies	Error bars are mean ± s.e.m.	0.5Hz: p = 0.068<br>1Hz: p = 0.026<br>*2Hz: p < 0.001*<br>*4Hz: p < 0.001*<br>*8Hz: p = 0.002*<br>16Hz: p = 0.019	0.5Hz: t(67) = 1.9<br>1Hz: t(68) = 2.7<br>2Hz: t(66) = 4.3<br>4Hz: t(64) = 4.8<br>8Hz: t(61) = 3.2<br>16Hz: t(51) = 2.4
	**B** right (blue)	Two-tailed Student's t-tests with Bonferroni correction for multiple comparison at each frequency (threshold p = 0.0083)	272	Phase values from regular otolith afferents in response to GVS or motion across different frequencies	Error bars are mean ± s.e.m.	*0.5Hz: p < 0.001*<br>*1Hz: p = 0.006*<br>*2Hz: p < 0.001*<br>*4Hz: p = 0.001*<br>*8Hz: p < 0.001*<br>16Hz: p = 0.151	0.5Hz: t(46) = 5.7<br>1Hz: t(46) = 2.9<br>2Hz: t(46) = 3.8<br>4Hz: t(45) = 3.4<br>8Hz: t(42) = 4.3<br>16Hz: t(35) = 1.5

8	**E**	Two-tailed Student's t-tests with Bonferroni correction for multiple comparison at each frequency (threshold p = 0.00625)	781	Log-normal transformed detection threshold values from 119 canal afferents (56 irregular versus 63 regular) across 8 frequencies	Error bars are mean ± s.e.m. of the non-transformed data	0.1Hz: p = 0.781<br>0.2Hz: p = 0.599<br>0.5Hz: p = 0.212<br>1Hz: p = 0.504<br>2Hz: p = 0.257<br>4Hz: p = 0.011<br>*8Hz: p = 0.0052*<br>*16Hz: p = 0.004*	0.1Hz: t(69) = 0.3<br>0.2Hz: t(70) = 0.5<br>0.5Hz: t(108) = 1.3<br>1Hz: t(111) = 0.7<br>2Hz: t(110) = 1.1<br>4Hz: t(106) = 2.6<br>8Hz: t(103) = 2.9<br>16Hz: t(88) = 3.0
	**E** (red)	Linear mixed model	378	Log-normal transformed detection thresholds from 56 irregular canal afferents across 8 frequencies	Error bars are mean ± s.e.m. of the non-transformed data	*p < 0.001 for frequency factor*	$F_{7,317.58}$ = 11.62 for frequency factor
	**E** (blue)	Linear mixed model	403	Log-normal transformed detection thresholds from 63 regular canal afferents across 8 frequencies	Error bars are mean ± s.e.m. of the non-transformed data	*p < 0.001 for frequency factor*	$F_{7,339.18}$ = 7.01 for frequency factor
	**F**	Two-tailed Student's t-tests with Bonferroni correction for multiple comparison at each frequency (threshold p = 0.00625)	553	Log-normal transformed detection threshold values from 84 otolith afferents (54 irregular versus 30 regular) across 8 frequencies	Error bars are mean ± s.e.m. of the non-transformed data	0.1Hz: p = 0.279<br>0.2Hz: p = 0.718<br>0.5Hz: p = 0.493<br>1Hz: p = 0.931<br>2Hz: p = 0.405<br>4Hz: p = 0.349<br>8Hz: p = 0.040<br>16Hz: p = 0.197	0.1Hz: t(47) = 1.1<br>0.2Hz: t(48) = 0.4<br>0.5Hz: t(78) = 0.7<br>1Hz: t(82) = 0.1<br>2Hz: t(78) = 0.8<br>4Hz: t(73) = 0.9<br>8Hz: t(74) = 2.1<br>16Hz: t(57) = 1.3
	**F** (red)	Linear mixed model	341	Log-normal transformed detection thresholds from 54 irregular otolith afferents across 8 frequencies	Error bars are mean ± s.e.m. of the non-transformed data	*p < 0.001 for frequency factor*	$F_{7,281.66}$ = 11.31 for frequency factor
	**F** (blue)	Linear mixed model	212	Log-normal transformed detection thresholds from 30 regular otolith afferents across 8 frequencies	Error bars are mean ± s.e.m. of the non-transformed data	*p < 0.001 for frequency factor*	$F_{7,175.49}$ = 5.12 for frequency factor
	**E/F** (red)	Two-tailed Student's t-tests with Bonferroni correction for multiple comparison at each frequency (threshold p = 0.00625)	719	Log-normal transformed detection threshold values from 110 irregular afferents (56 canals versus 54 otoliths) across 8 frequencies	Error bars are mean ± s.e.m. of the non-transformed data	0.1Hz: p =0.413<br>0.2Hz: p = 0.583<br>0.5Hz: p = 0.722<br>1Hz: p = 0.398<br>2Hz: p = 0.983<br>4Hz: p = 0.287<br>8Hz: p = 0.902<br>16Hz: p = 0.252	0.1Hz: t(55) = 0.8<br>0.2Hz: t(57) = 0.6<br>0.5Hz: t(85) = 0.4<br>1Hz: t(86) = 0.9<br>2Hz: t(84) = 0.0<br>4Hz: t(84) = 1.1<br>8Hz: t(81) = 0.1<br>16Hz: t(67) = 1.2

	**E/F** (blue)	Two-tailed Student's t-tests with Bonferroni correction for multiple comparison at each frequency (threshold p = 0.00625)	615	Log-normal transformed detection threshold values from 93 regular afferents (63 canals versus 30 otoliths) across 8 frequencies	Error bars are mean ± s.e.m. of the non-transformed data	0.1Hz: p = 0.777<br>0.2Hz: p = 0.588<br>0.5Hz: p = 0.476<br>1Hz: p = 0.514<br>2Hz: p = 0.974<br>4Hz: p = 0.920<br>8Hz: p = 0.992<br>16Hz: p = 0.699	0.1Hz: t(61) = 0.3<br>0.2Hz: t(61) = 0.5<br>0.5Hz: t(101) = 0.7<br>1Hz: t(107) = 0.7<br>2Hz: t(104) = 0.0<br>4Hz: t(95) = 0.1<br>8Hz: t(96) = 0.0<br>16Hz: t(78) = 0.4
